# Improving Night Time Driving Safety Using Vision-Based Classification Techniques

**DOI:** 10.3390/s17102199

**Published:** 2017-09-24

**Authors:** Jong-Chih Chien, Yong-Sheng Chen, Jiann-Der Lee

**Affiliations:** 1 Degree Program of Digital Space and Product Design, Kainan University, Taoyuan City 338, Taiwan; jcchien@knu.edu.tw; 2 Department of Electrical Engineering, Chang-Gung University, Taoyuan City 333, Taiwan; KK770102@hotmail.com; 3 Department of Neurosurgery, Chang Gung Memorial Hospital, LinKou, Taoyuan City 333, Taiwan; 4 Department of Electrical Engineering, Ming Chi University of Technology, New Taipei City 24301, Taiwan

**Keywords:** MSR, KAZE, CNN, BoF, driver safety, advanced driver assistant

## Abstract

The risks involved in nighttime driving include drowsy drivers and dangerous vehicles. Prominent among the more dangerous vehicles around at night are the larger vehicles which are usually moving faster at night on a highway. In addition, the risk level of driving around larger vehicles rises significantly when the driver’s attention becomes distracted, even for a short period of time. For the purpose of alerting the driver and elevating his or her safety, in this paper we propose two components for any modern vision-based Advanced Drivers Assistance System (ADAS). These two components work separately for the single purpose of alerting the driver in dangerous situations. The purpose of the first component is to ascertain that the driver would be in a sufficiently wakeful state to receive and process warnings; this is the driver drowsiness detection component. The driver drowsiness detection component uses infrared images of the driver to analyze his eyes’ movements using a MSR plus a simple heuristic. This component issues alerts to the driver when the driver’s eyes show distraction and are closed for a longer than usual duration. Experimental results show that this component can detect closed eyes with an accuracy of 94.26% on average, which is comparable to previous results using more sophisticated methods. The purpose of the second component is to alert the driver when the driver’s vehicle is moving around larger vehicles at dusk or night time. The large vehicle detection component accepts images from a regular video driving recorder as input. A bi-level system of classifiers, which included a novel MSR-enhanced KAZE-base Bag-of-Features classifier, is proposed to avoid false negatives. In both components, we propose an improved version of the Multi-Scale Retinex (MSR) algorithm to augment the contrast of the input. Several experiments were performed to test the effects of the MSR and each classifier, and the results are presented in experimental results section of this paper.

## 1. Introduction

The ultimate goal of our study is to increase driver’s safety by alerting the driver when driving under non-ideal conditions using a vision-based ADAS. Advanced Drivers Assistance (ADAS) is slowly reaching technological maturity. There are already many ADAS models that use the video driving recorder as a sensor, for example, for detecting pedestrians [1]. A few models add a Forward Collision Warning (FCW) radar [2], as well as other collision avoidance systems [3] such as the intelligent reversing radar systems to warn the driver while moving. However, while not every vehicle is equipped with an expensive radar detection system, most cars are equipped with video driving recorders which can readily supply video input for processing.

The danger level of driving any vehicle in non-ideal environments is greatly raised when the driver’s attention level is sub-optimal [4,5,6,7,8,9,10]. The video output of video driving recorders can be processed in a video-based ADAS system to help the driver in these situations. In this paper, we propose a system that combines a module to determine the level of driver’s drowsiness with a module to detect the presence of large vehicles. The drowsiness module operates by detecting and tracking the driver’s eyes using infrared images. The large vehicle detector module detects and tracks large vehicles via input from a driving recorder. These modules use classifiers based on different features, including CNN-based features [11], and a Bag-of-Features (BoF) [12] classifier that uses the patent-free KAZE/AKAZE features.

In previous studies for detecting drowsiness using the driver’s eyes [5,6,7,8,9,10], better results were obtained under better lighting conditions, such as daylight. However, in studies of night-time detection using infrared images, various works report an average detection accuracy of 92.05% [9], 92.21% [8], and up to 94.74% [10] (without validating the eyes during tracking). Flores, et al. [10] used a more sophisticated method to obtain this result, where difference, edge, and radial-transformed information were preprocessed with a Gabor filter before being used to train an SVM classifier. In our drowsiness component, we used a much simpler method. We used a version of MSR to improve the image contrast before detecting and tracking the pupils in the driver’s eyes using a simple heuristic so that drowsiness detection in real-time could be attained. 

**Figure 1:**
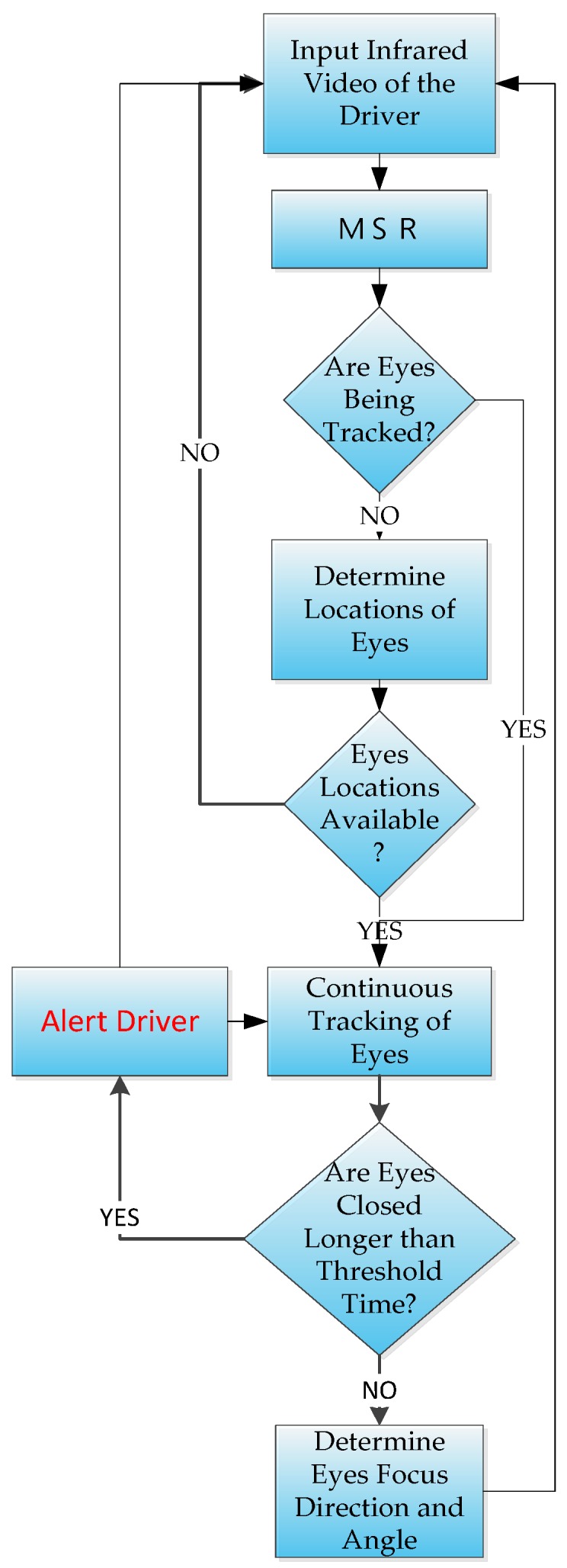
The flowchart for the driver attention component.

Experimental results using six videos of five different drivers show that we could achieve an average accuracy of 94.26%, which is comparable to the results of previous studies. In previous studies for detecting vehicles at night [13,14], cameras were set up in a fixed location above the vehicles and used to locate the head and tail lights of the surrounding vehicles. However, their purpose was to count the passing vehicles [13], or identify license plates [14], which differs from the purpose and method of our study. Our study uses the video driving recorder onboard the vehicle itself to identify the presence of vehicles ahead, and to differentiate large vehicles from other vehicles. Our study seeks to determine the existence of situations at night when the driver should be alerted in real-time. These situations include the driver’s lowering of attention or drowsiness; i.e., not in a sufficiently wakeful state to process warnings, and when the driver’s vehicle is in the vicinity of large moving vehicles. These situations are determined using two different components. The driver’s attention-level detector component uses the images from a driver-facing infrared camera as input, then a version of the MSR function is applies to improve its contrast before processing. A trained LBP-based Adaboost classifier to determine the initial locations of the eyes, then a tracking algorithm [15] is used to track the eyes continuously. The areas in the images containing the eyes are used to determine whether the pupils are present. If the pupils cannot be located in either eye then it is assumed that the driver is either looking downward or has his/her eyes closed due to drowsiness. If the pupils cannot be detected for longer than a preset threshold, then a warning would be issued. The flowchart for this component is shown in Figure 1.

**Figure 2:**
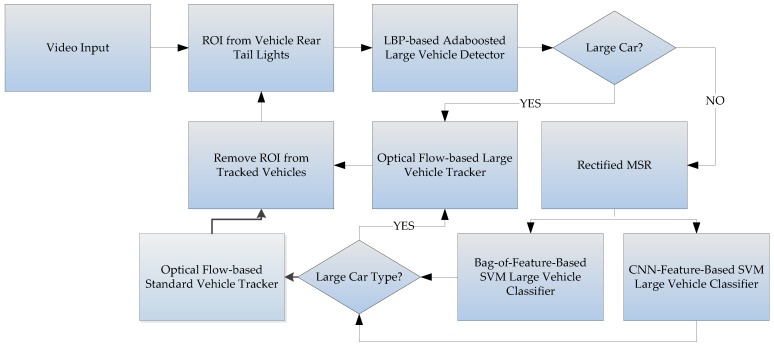
The flowchart for the large vehicle detector.

The large vehicle detector component first uses the rear-light detection method proposed by Wu and Lee [16] to find the locations of rear car lights within a video frame. Then the lights are paired using image mirroring, and the areas between paired lights are the initial guesses for the regions-of-interest [17]. These regions are assumed to contain individual vehicles within them. The contents of these regions are then passed to a bi-level classifier system for identification. The first level of the classifier system is a trained Adaboost classifier [18], and the second level includes a novel KAZE-based BoF classifier, and a CNN-features-based classifier are used to catch the false negatives. The bi-level classifier system is used in the following way: first, the images within each region are passed to a trained LPB-based Adaboost classifier in order to quickly determine whether it contains a large vehicle, and the positives are tracked immediately. The regions determined to be negatives by the first classifier are then passed through a rectified version of MultiScale-Retinex (MSR) based on [19] to improve the contrast before being re-classified by the second-level classifiers. The second-level classifiers include a KAZE/AKAZE [20] features-based Bag-of-Features classifier and a Convolution-Neural-Network features-based classifier. Depending on the number of regions-of-interest and the contents within the regions, either or both classifiers would be used in order to further separate true negatives from false negatives. If either the KAZE-based classifier or CNN-based classifier [21,22,23,24,25] detects a false negative, only then is this information used to update the tracking algorithm, so the classification operations would not disrupt the tracking algorithm. Our experiments shows that preprocess the regions using the rectified MSR can improve the detection rate of the KAZE detector, even under bad conditions. The system process chart is illustrated in Figure 2. The true negatives are assumed to be standard-sized vehicles, and can be tracked separately.

## 2. Driver Attention Detection

**Figure 3:**
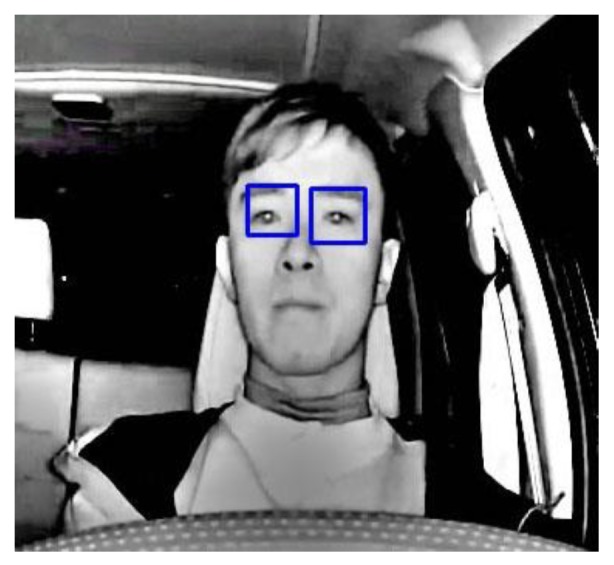
Detection and tracking of the driver’s eyes.

An infrared camera is chosen to capture driver’s upper body image during night-time driving. The condition of the eyes are used to determine the level of focus attention of the driver. After an MSR operation is used to improve the contrast of the image, a trained LBP-based Ada-boost classifier is then applied to do a quick initial detection of the locations of the eyes; the requirement for this stage of processing is that the eyes must be fully opened and the driver is facing directly ahead, as shown in Figure 3. Once the eyes are detected, they are passed to a tracking algorithm [15] that tracks their location changes between frames.

**Figure 4:**
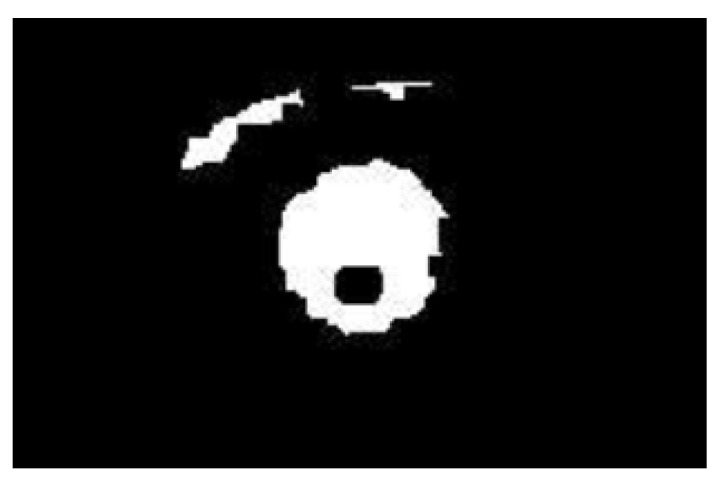
Detection of the infrared reflection in the pupil.

The regions containing the eyes are then passed to a pupil detection algorithm, which seeks to determine whether the driver’s eyes are open. This is done by using a heuristic of first inverting the video within these regions then thresholded so that reflection of the infrared lights reflected by the pupils would appear as black surrounded by white pixels, as shown in Figure 4, and can then be easily located. 

**Figure 5:**
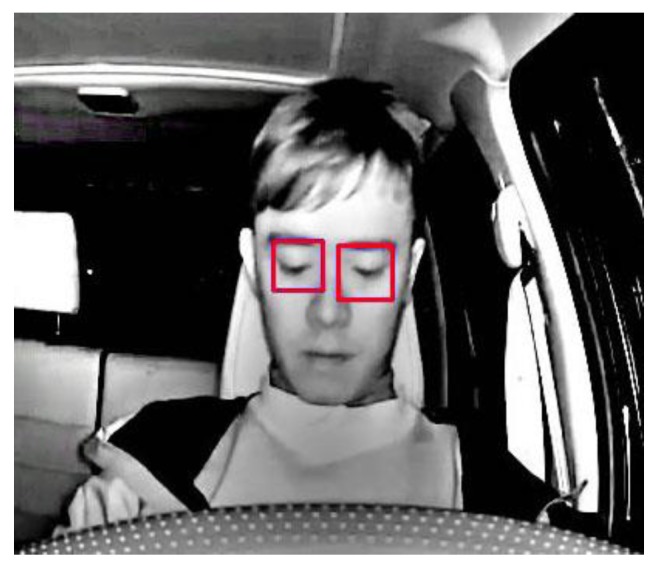
Failure to detect pupils.

If the heuristic fails to detect both pupils, as shown in Figure 5, for longer than a preset number of frames, the algorithm would alert the driver by issuing a warning in order help him or her regain focus. In Figure 5, the eyes are marked as red to indicate that the pupils could not be detected.

**Figure 6:**
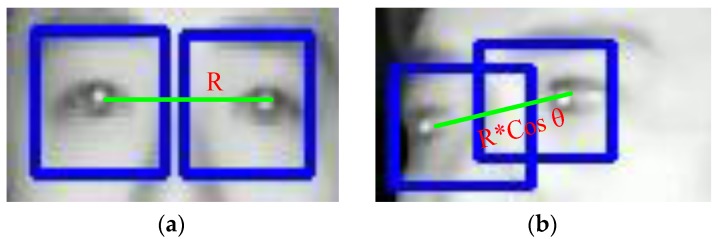
Distance between eyes: (**a**) eyes facing front (**b**) face rotated by angle θ.

However, if the driver’s pupil can be detected, then the locations and the distance between the detected eyes and the distance between them are used to calculate the relative angle of focus of the driver, as shown in Figure 6, where *R* represent the distance between the eyes when facing front, and θ approximates the angle the head rotation. The rotation angle θ, is approximated by the following equation:

(1) $$\theta =\mathrm{arcsec}\left(\frac{R}{R\cos\left(\theta \right)}\right)$$

where *R* is the distance between the eyes when the driver’s head is facing toward the camera, and *R*cos(θ) is the distance between eyes measured by the camera image when the head is turned away from the camera.

**Figure 7:**
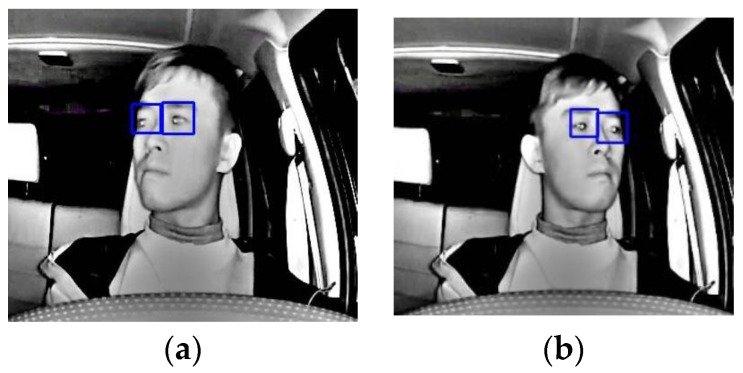
Determining the angle-of-focus: (**a**) focus to the right of driver (**b**) focus to the left.

This information can be helpful to determine if the driver’s attention is on the road ahead. In Figure 7, the tracking of driver’s eyes when the driver’s head is rotated are shown. In order to test the detection accuracy, a total of five infrared video sequences of four different drivers were used to test our heuristic, and an average accuracy of 94.26% was achieved. That is, 94.26% of the times the instances where the driver’s eyes are closed or when the eyes are opened, are correctly identified. The individual results will be shown in the experimental results section. This value is comparable to the average of around 92% (without validating the eyes during tracking) obtained by [8,9], and 94.7% obtained by [10] using a more sophisticated method under similar conditions.

## 3. Classifiers

**Figure 8:**
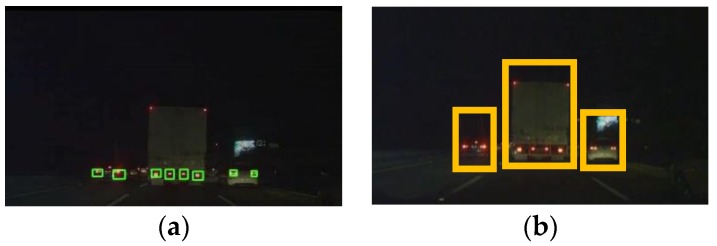
Locating Regions-of-Interest (**a**) taillights location (**b**) initial estimations of ROI.

As shown by the flowchart in Figure 2, the rear car light detection algorithm proposed by Wu and Lee [16] is used, where regions surrounding detected rear tail lights are extracted, then mirror-matching is used to pair the rear lights. If a match is successful, then it is assumed that this pair of tail-lights belongs to the same vehicle, and the body of the vehicle is between the two matched tail-lights. If this assumption holds true, then the distances between the tail-lights can be used to approximate the width of the vehicle between. Each region-of-interest is extracted by using an initial assumption that each vehicle is a large vehicle, as shown in Figure 8, so the height of each region vs. the width of each region would be proportional to that of an average large vehicle. The content of each region is immediately passed to a trained LBP-based Adaboost classifier to quickly determine whether the content actually is a large vehicle or not. Figure 8 shows three paired regions, where each region is first assumed to contain a large vehicle. The content of each region would be quickly determined as a large or small vehicle using a trained LBP-based Adaboost classifier, then separately tracked.

This results in an initial estimation are the locations of large vehicles. However, because false negatives are possible, in order to catch these false negatives, a rectified MSR based on [19] is used first to improve the contrast before passing each region to the second-stage classifiers. These second-stage classifiers use SVM’s trained on extracted features to catch the false negatives. The reason they were not considered for the first stage is because they take longer time to process than the Adaboost classifier. Their processing time includes the time for detecting and extracting features plus the classification time. These second-stage classifiers include a classifier that uses the patent-free KAZE/AKAZE [20] feature descriptors using bag-of-features method, and a classifier that uses a trained Convolutional Neural Network-extracted feature descriptors. We performed several experiments to test the performance differences between the KAZE and the AKAZE BoF classifiers, results of which will be discussed in the experimental results section. The results show that the KAZE detector locates more feature points than the AKAZE detector in the night scenes, but takes longer to process. However, in terms of classification accuracy, the KAZE BoF classifier outperforms the AKAZE BoF classifier. For the CNN-features-based classifier, we chose the ImageNet-trained AlexNet model [21,25] as the base structure for building the classifier. This classifier uses the outputs from a fully-connected layer before the softmax output layer as features for training and classification. Our experimental results show that an SVM trained using the CNN-features, with some exceptions, is generally more accurate than the KAZE or AKAZE BoF classifiers.

### 3.1. Rectified Multi-Scale Retinex (MSR)

**Figure 9:**
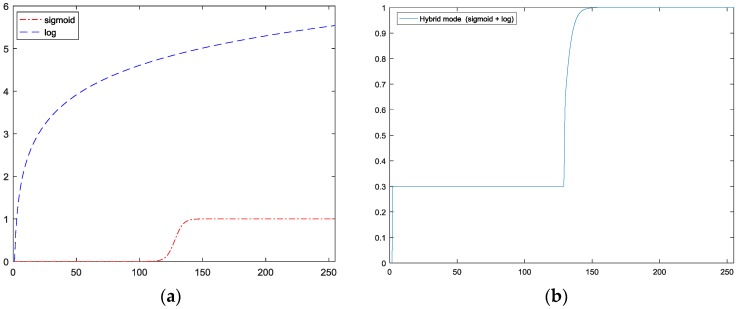
(**a**) Sigmoid function vs. standard Log function (**b**) our Sigmoid function.

For the purpose of improving the contrast for nighttime driving video, the Sigmoid function is used to replace the Log function in the original MSR algorithm, in order to minimize overexposure. The original MSR function can be written as:

(2) $$O\left(x,y\right)=\overset{N}{\underset{n=1}{\sum }}W_{n}\left\{\mathrm{Log}\left[I\left(x,y\right)\right]-\mathrm{Log}\left[I\left(x,y\right)M_{n}\left(x,y\right)\right]\right\}$$

where *W* is the weighting function, *I* is the input image, and *M* is the convolution mask. The inner expression can be written as:

(3) $$\log\left[\frac{I\left(x,y\right)}{I\left(x,y\right)M_{n}\left(x,y\right)}\right]$$

which can be understood as the ratio between the current pixel and its weighted neighbors. However, as can be seen in Figure 8, where the Log function grows quickly, and easily reach beyond the original range of image values, while the Sigmoid function remain within the range of image value. The Sigmoid function we implemented is:

(4) $$\mathrm{sig}\left(x\right)=\frac{1}{1+e^{-k\left(x-1\right)}}$$

where *k* is used to control the shape of function. Because the Log function quickly climbs beyond the value of 1 as shown in Figure 9a, for night scenes processed using the Log-based MSR can be over-exposed, resulting in significant loss of data. By contrast, a Sigmoid-based MSR, because its upper cut-off is at 1, so it is considered to be a rectifier, which could minimize the loss of data. However, the standard Sigmoid function quickly reaches the lower cut-off at 0, has the effect of losing critical information, when ratio in the region is low, so we choose to suppress this effect by modifying the MSR by replacing its values of Sigmoid function with the values of Log function values when the ratio falls below 0.3, as shown in Figure 9b. The value of 0.3 was obtained experimentally.

The weighting function we used is:

(5) $$W\left(x,y\right)={\left(I\left(x,y\right)*G\left(x,y\right)\right)}^{a}$$

where ∗ is the operator for convolution, *G* is the Gaussian function, and *a* is used to control the weighting. Finally the result after MSR is combined with the original image for the final result *X*:

(6) $$X\left(x,y\right)=O\left(x,y\right)\cdot W_{t}\left(x,y\right)+I\left(x,y\right)\cdot \left[1-W_{t}\left(x,y\right)\right]$$

where *W_t_* is the weight used to combine the images.

**Figure 10:**
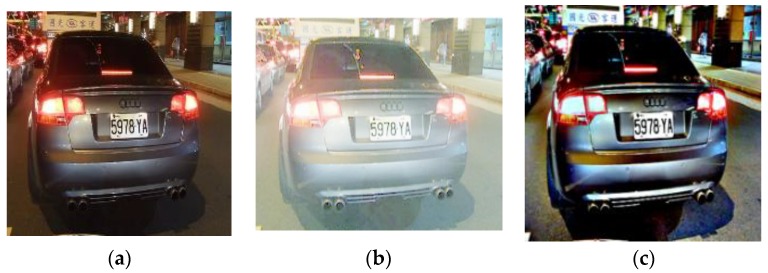
Comparison between different MSR results (**a**) Original, (**b**) Log-based MSR, (**c**) Sigmoid-based MSR.

Figure 10 compares the original night image, which has low contrast; with images preprocessed using Log-based MSR and our Sigmoid-based MSR. As can be seen, the image processed with the Log-based MSR in Figure 9b looks overexposed compared to the image processed using our Sigmoid-based MSR in Figure 9c. For testing its classification efficacy we performed an experiment and applied both Log-based MSR and Sigmoid-based MSR to 200 images and measured the classification rate of KAZE-BoF-based and AlexNet CNN features-based classifiers in classifying these 200 images. Based on the results of these experiments, we can conclude our Sigmoid-based MSR can help the performance of these two types of classifiers, especially when the ratio of training data to overall data exceeds 50%. The details of this experiment will be presented in the experimental results section.

### 3.2. Convolutional Neural Network (CNN) Features

**Figure 11:**
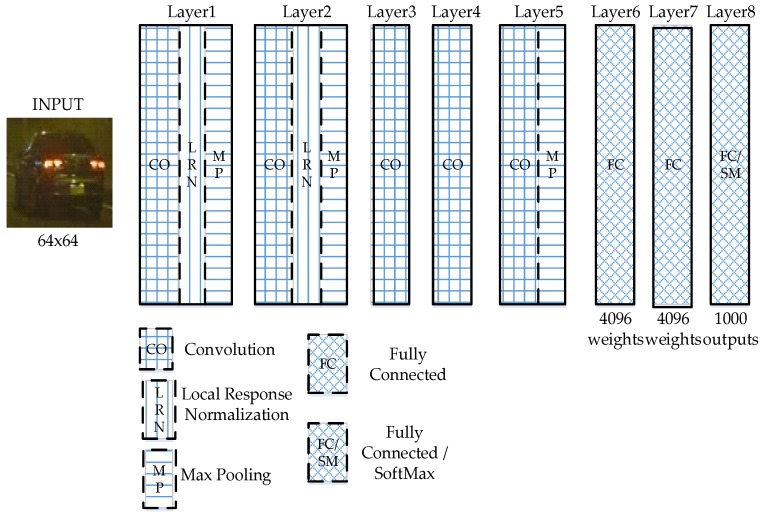
AlexNet Layers.

A convolutional neural network is composed of one or more convolution layers plus fully connected layers (corresponding to classical neural networks), but also include weighting and pooling layers. This structure allows convolutional neural network to make use of: (1) nonlinear units (ReLU), (2) dropouts that are used during training to selectively dismiss individual neural unit in order to avoid overfitting the model, and (3) overlap pooling [22] in order to maximize pooling and reduce the averaging effect of average pooling. Our CNN-base feature classifier makes use of features taken from the second to the output softmax layer, FC7, of AlexNet, shown in Figure 11 [24], as training and testing features for an SVM classifier. Features taken from a deeper layer tend to result in better classification performance than those taken from a shallower layer. This is the process of our CNN-based classifier: The content of a region-of-interest after MSR is used as input into the ImageNet-trained AlexNet, then the output features are extracted from FC7 [23] layer, and are used to train a multi-class SVM classifier.

We performed an experiment to select a suitable CNN structure to extract feature for our purposes before choosing the AlexNet CNN structure. The first experiment compares the performance of the features from two pre-trained CNN architectures using the ImageNet data: the VggNet [26] architecture and the AlexNet [26] architecture in classifying images of night-time vehicle images. We used a total of 23,066 images of large and small vehicles started at 90% for training (10% for test), and slowly decreased the ratio of training data until the features from one of the structure gave us an error in classification. Using this process, we finally chose AlexNet. Another reason for selecting AlexNet was results of [25], where AlexNet was shown to be faster than most of the other ImageNet-trained CNNs. Figure 11, based on a figure in [24], presents a simple to understand structure for the AlexNet CNN. The AlexNet structure includes five convolutional layers and sublayers in total. There are two normalization sublayers following the first two convolutional sublayers, three max pooling sublayers, follows by three fully-connected layers, and the last fully-connected layer is a softmax layer which outputs CNN classification results. The first two fully-connected layers each has 4096 outputs, and the softmax layer outputs onto 1000 classes, according to ImageNet data. Our systems takes the outputs from FC7 and used them to form 4096-long feature vectors as training vectors for a multi-class SVM.

A second experiment was designed to test whether using other classification features that also calculates the features from the entire image region, such as the Hog and the LBP features, would perform better than the AlexNet CNN-based features. The experiment we performed used only 486 of the 23,066 images and using features such as LBP [26], HoG [26] and AlexNet CNN [21,25] in testing their effectiveness in classification using SVM, and the clear winner was the AlexNet CNN. The results of this experiment will be discussed in the experimental results section.

### 3.3. KAZE/AKAZE Features-Based BoF Classifier

The patent-free KAZE feature, introduced by Alcantarilla et al. [20], uses the Addition Operator Split (AOS) algorithm and nonlinear diffusion, as shown in Equation (7), to build a nonlinear scale space, from which sampled values would be closer to the original values than those taken from a linear scale space:

$$\frac{\partial L}{\partial t}=\mathrm{divergence}\left(c\left(x,y,t\right)\cdot \nabla L\right)$$

where:

(7) $$c\left(x,y,t\right)=g\left(\left|\nabla L_{\sigma }\left(x,y,t\right)\right|\right)$$

where:

$$g=\frac{1}{1+\frac{{\left|\nabla L_{\sigma }\right|}^{2}}{\lambda^{2}}}$$

where *L* is the luminance, *L_σ_* is the Gaussian-smoothed *L*, and *λ* is the contrast factor. The AKAZE features seeks to improve the speed of the KAZE by trying to calculate the scale space faster. The difference between KAZE and AKAZE descriptors from the patented SURF and SIFT descriptors is that KAZE and AKAZE calculate the main direction of the feature point for building nonlinear scale space.

**Figure 12:**
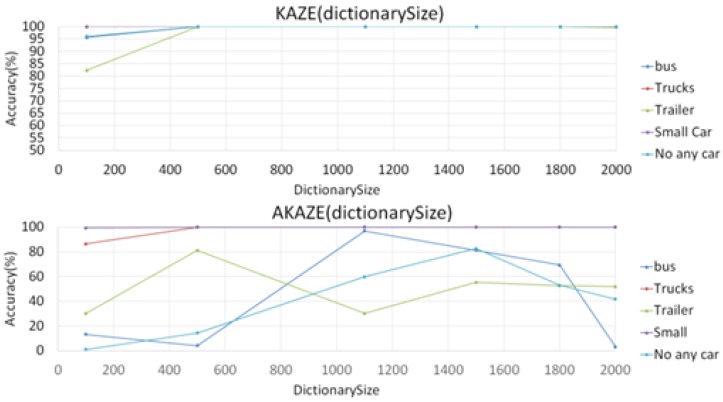
KAZE/AKAZE BoF Classification Accuracy vs. Dictionary Size.

Unlike the CNN-based features, KAZE and AKAZE features have to perform key-points detection first before extracting feature descriptions in each detected key-point in the sample space. They extract disordered sets of local small blocks from which descriptors are calculated from the key-points. These descriptors are then grouped into local clusters using a clustering algorithm, such as the K-means algorithm, where each cluster center is treated as a vocabulary word for a visual dictionary, and a term characterized by a clustering center corresponds to the formation of a code word. However, KAZE is relatively slow due to the calculation for nonlinear scale space, but the AKAZE feature is faster. We performed experiments to test KAZE against AKAZE by using them as features in BoF classifiers in the task of classifying large vehicle types using different dictionary sizes (number of bags in BoF). The purpose of these experiment was to determine an appropriate dictionary size for both KAZE and AKAZE BoF classifiers. However, as the graphs in Figure 12 show, the results not as unexpected. 

Figure 11 shows that the dictionary size has relatively little effect on the KAZE features from 500 onward, while it greatly impacts the performance of the AKAZE features, so a single value for dictionary size that would work for the AKAZE BoF classifier could not be easily found; we finally settled on 1500 as default. Also, in another experiment in classifying night-time vehicle images, we found that the performance of the AKAZE features could not match those of KAZE, due to the fact that the AKAZE feature point detector detects far fewer key points than those of the KAZE detector. This will be discussed in the experimental results section. So from the result of these experiments, it appears that the KAZE-based BoF classifier would be a better choice than the AKAZE-based BoF classifier.

## 4. Experimental Results and Discussion

A total of eight experiments were set up to test the performance and accuracy of these two modules. The first five experiments were set up to test the large vehicle detector module according to the order of the flowchart in Figure 1. In the first experiment, the LBP-based Adaboost Classifier was tested, followed by the second experiment to test the rectified MSR; then the third, which is to test the BoF classifiers; followed by the fourth, which is to compare the CNN-based classifier against similar features; then the fifth, which is a comparison experiment between the BoF and the CNN-based classifiers in when the input images are crisp and cleanly extracted. The next two experiments sought to test the same classifiers when the input images were not crisp and cleanly or even correctly extracted. The last experiment is set up to test the accuracy of the driver attention module. The computer we used for the experiments had the following configuration: Intel i7 3630 M CPU (base frequency of 2.4 GHz, four cores), 8 GB Ram, running only on the CPU without GPU enhancement. 

The first experiment seeks to test the performance of the LBP-based Adaboost classifier, which is the first-pass classifier according to the flowchart for the large vehicle detector module. The positive training samples include several types of large vehicles, such as buses, trucks and trailers, for a total of 10,420 positive images. The negative training samples include images of small sedans and empty street lights, for a total of 12,646 images, so the total of number images is 23,066 images. Two LBP-based Adaboost classifiers were trained, one to the 21st stage, and the other to only the 20th stage. Table 1 shows the results comparing these two versions and shows a great difference with just one single stage added. We decided to use the 21-stage LBP-based Adaboost classifier.

The second experiment sought to test the effectiveness of our Sigmoid-based MSR vs. the traditional Log-based MSR as preprocessors when used in the second-stage classifiers. We used a total of 200 images of large and small vehicles taken at night. 

**Figure 13:**
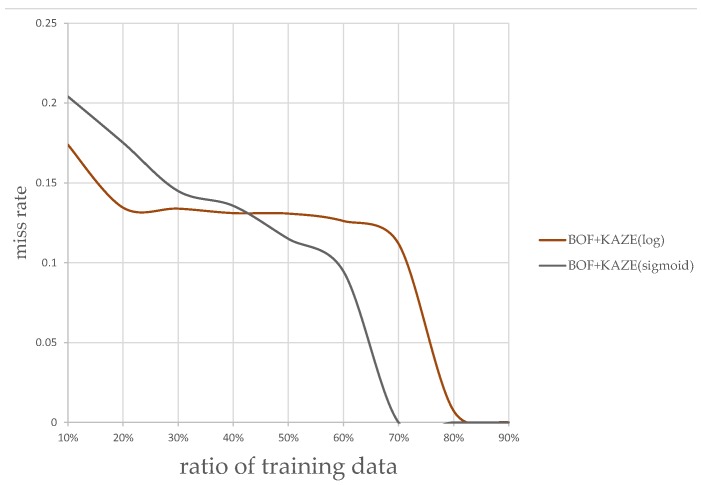
Kaze-based BoF classification rates.

**Figure 14:**
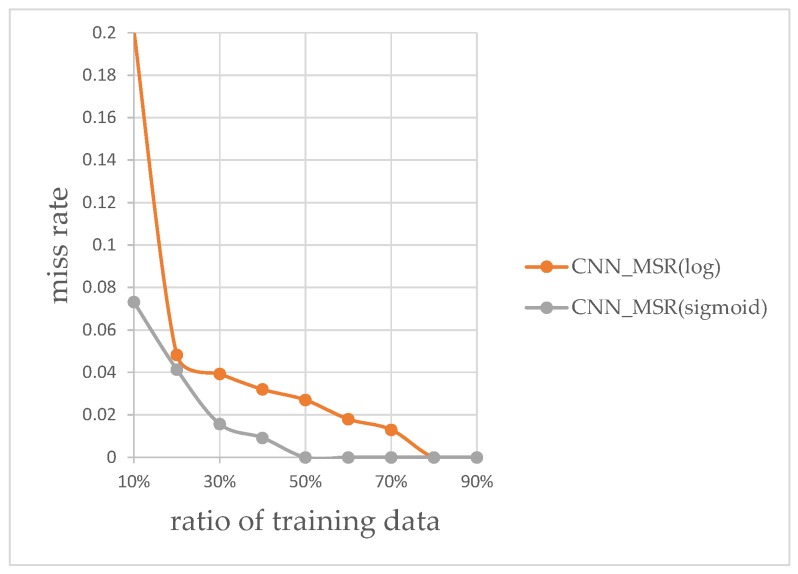
CNN features-based classification rates.

The plots of classification miss rate vs. ratio of training data to overall data are generated for the classifier using the AlexNet CNN-based features, and the classifier using KAZE features. Figure 13 and Figure 14 show the plots using the KAZE-based BoF and CNN-based classifiers, respectively. As can be seen in Figure 13, our Sigmoid-based MSR markedly improved the performance of the KAZE-based BoF classifier once the ratio of training data to overall data passed 45%. For the CNN features-based classifier, as shown in Figure 14, the Sigmoid-based MSR outperformed the Log-based MSR in almost every instance. This experiment shows that our Sigmoid-based MSR can be of help for both types of classifiers and validated our design.

The third experiment sought to test the effectiveness of the BoF classifier using the KAZE vs. the AKAZE features. We first separated the training set into four classes: bus, truck, trailer, and standard-sized sedan. The training set include 8532 bus images, 1466 truck images, 352 trailer images, and 3117 sedan images, for a total of 13,467 images. Table 2 shows the classification results using KAZE and AKAZE features-based BoF classifiers when 80% of all data were used as training data. The results show that the AKAZE feature is less accurate than the KAZE features when used in BoF classifiers for these images.

In the fourth experiment we wanted to validate our choice of using CNN-features for the classifier, so we compared the AlexNet CNN-features against similar feature-based types that also calculate features using the entire image, such as the HOG and LBP [26] features. In the setup for this experiment, all the images were preprocessed using the rectified MSR before the calculations for the feature vectors. These feature vectors are then passed to respective SVMs for training and classification. In this experiment, because we were interested only in relative accuracy, only 486 images were selected for training and classification. These images were all resized according to the requirement for AlexNet, which is 64 by 64. Table 3 shows the number of features extracted by CNN, HOG and LBP feature extractors for each of the resized images.

**Figure 15:**
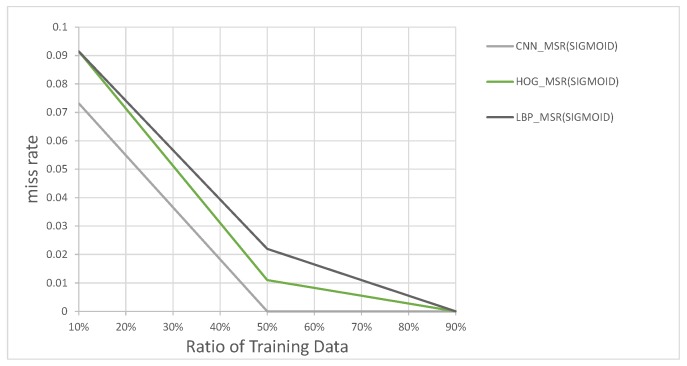
Classification miss rates for CNN, HOG and LBP.

These feature vectors are then used to train each SVM for classifying large and small vehicles at the ratios of 10%, 50% and 90% of all data as training data. Figure 15 shows the results of this experiment. As seen in Figure 15, the classifier using the LBP feature vectors, which are the longest feature vectors of the three, performed the worst. The HOG features classifier was the second-best performer, and CNN-based classifier was the best performer overall, so we decided to use CNN, as it performed the best of all three according to our needs.

In the fifth experiment, we used the images from the third experiment plus 10% additional images taken during sub-ideal conditions, such as during rainshowers, and preprocessed each one with our proposed Sigmoid-based MSR. Each of these images is a cleanly cropped image containing only a single vehicle. These images then used to compare the performance of classification of the CNN features-based classifier against the KAZE features-based and AKAZE features-based BoF classifiers in separating large vehicles from the small vehicles. The processing time was taken for each image in order to calculate the average execution time to process each image. The average processing time are decomposed into the time used for feature extraction, the time used for SVM training, and the time used for SVM classification. The average processing time, in seconds, for each classifier are listed in Table 4. As can be seen in Table 4, the KAZE-based BoF takes the longest time on average. If we choose to keep both type of classifiers, then this result could imply that if the number of regions-of-interest is above a certain threshold, which would take longer time to process, then the use of CNN features-based classifier should take precedent over the KAZE-based classifier, but this is for future research.

**Figure 16:**
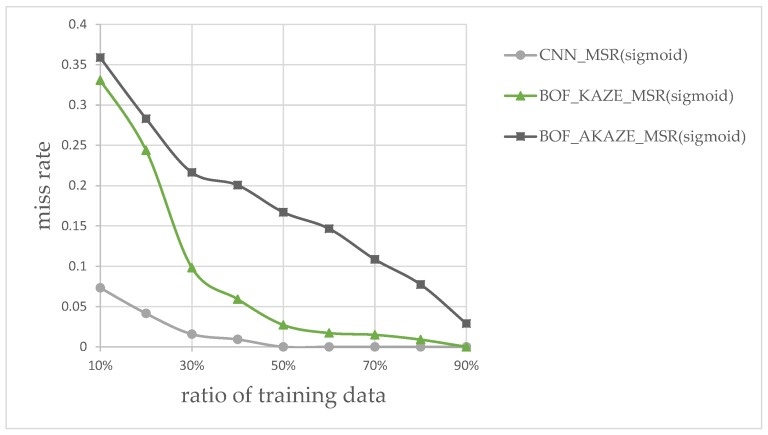
Miss rates vs. training data ratio for classifiers.

In addition to the processing time, the classification accuracy is also determined for each combination of MSR and classifier in this experiment. We measured the performance by splitting the images into different ratios of training and testing data; from 10% to 90% of images were allocated to training, and the rest for testing. The results for different ratios are shown in Figure 16. According to plot in Figure 16, the combination of using the rectified Sigmoid-based MSR and the CNN features-based classifier shows the best performance overall followed by the KAZE BoF then the AKAZE BoF. The experimental results, so far, shows that the CNN-based classifier appears to be sufficient without the BoF classifiers. We wished to find out, experimentally, whether there are situations where the KAZE feature, which requires detecting feature key-points, can perform better than the CNN-features-based classifier.

**Figure 17:**
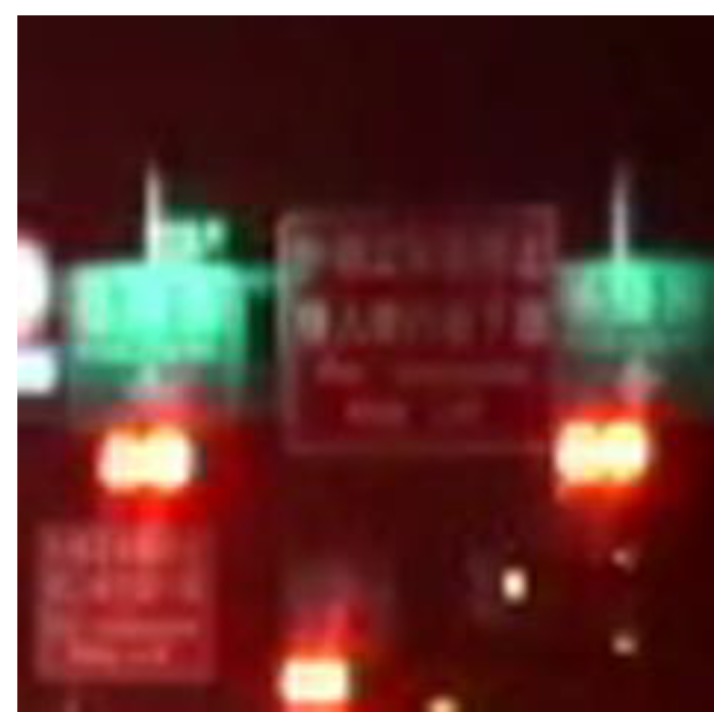
A traffic sign resembling vehicle tail-lights.

**Figure 18:**
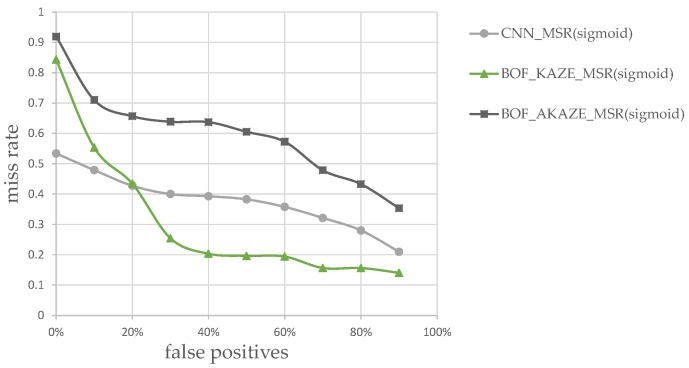
Vehicle classification miss rates vs. false positives.

Therefore, in the sixth experiment, we deliberately chose images that resemble vehicles but actually were street lights and signs. These images, an example of which is shown in Figure 17, are used to classify and generate the plots of miss rate vs. false positives (i.e., misidentified as large vehicles) for the CNN, KAZE and AKAZE classifiers. They are preprocessed using our Sigmoid-based MSR before classification training and testing. The results are shown in Figure 18, where the *y*-axis is the miss rate, and the *x*-axis are simply the percentage of the total number of images mis-classified as large vehicles by the classifiers.

**Figure 19:**
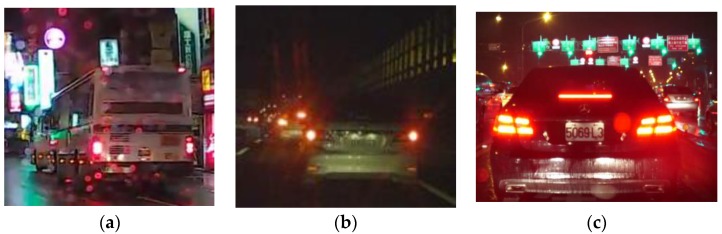
Vehicles not clearly defined (**a**) large cars with red signs (**b**) overlapping small cars (**c**) small car with signs.

These images were not in the training set of these classifiers, since they are neither large vehicle types nor sedans. Again, these images were first pre-processed with Sigmoid-based MSR before training and classification. As it can be seen in Figure 18, the miss rates of the KAZE-based classifier dropped faster than either CNN or AKAZE classifier, which implies that the KAZE-based features can perform better than CNN-features for easy-to-misidentify images. In order to test this hypothesis, we designed the seventh experiment, where the training and test images contain not just cleanly cropped vehicles itself but also contain signs and/or other vehicles. We envision that this type of situation can occur if the initial detection and pairing of tail-lights did not perform as expected, or the initial ROI extraction contains non-vehicular lights. Examples of these images are show in Figure 19. These images were not in the training set of the classifiers. Again, they were first pre-processed using the rectified MSR.

**Figure 20:**
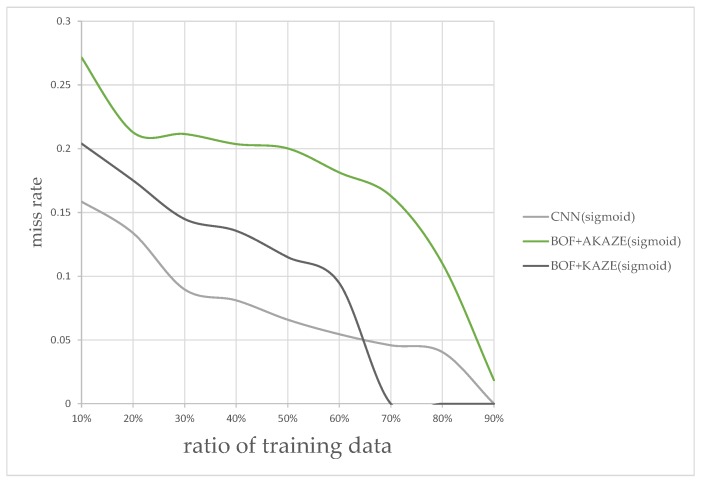
Classification miss rates vs. ratio of training data for sub-optimal images.

Figure 20 shows the miss rate vs. ratio of training data for this type of images only, at different training ratios. It is clear that the CNN-features-based classifier performed better for the ratios between 10% and 60%, but then the KAZE-based classifier shows better performance for the ratios after 65%. The result of this experiment shows that it would be wise to keep the KAZE-based classifier in our flowchart as a backup to catch the false-negatives.

**Figure 21:**
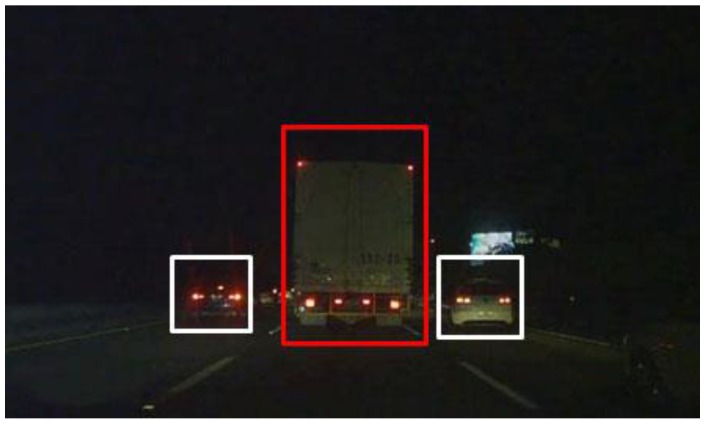
Regions for tracking after classification.

The results of the above experiments confirmed our flowchart for the large vehicle detector module. After the initial detection and extraction of tail-lights, the size of each region-of-interest is determined by assuming a large vehicle would be contained within each pair of tail-lights. This assumption could either result in a clean bounding box containing just the vehicle itself, in which case the Adaboost LBP classifier helps in the identification, and the false negatives can be caught using the CNN-features based classifier. However, if the bounding box contains other misleading features, such as those in Figure 19, then the KAZE-based classifier can help re-adjust the type of vehicle being tracking if the false negatives are not caught by the CNN-based classifier. The tracking of the vehicles by size is a separate process from the operations of the secondary classifiers, the purpose of which is to catch the false negatives, so tracking of vehicles is continuous unless false negatives are caught by the secondary classifiers. In which case, the trackers tracking the false negatives would be updated so they would know that they are tracking large vehicles rather than standard-size sedans, as shown in Figure 21, where the tracking of large vehicles and small vehicles types are tracked and marked separately.

**Figure 22:**
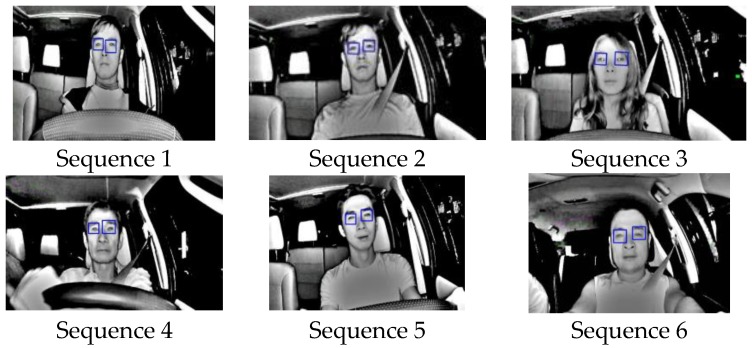
Six Test Video Sequences for Driver Drowsiness.

The last experiment was designed to test the accuracy of the driver drowsiness detection system. Six infrared video sequences taken at 20 fps of five different drivers were obtained, as shown in Figure 22, and their processed results were counted and displayed in Table 5. The purpose of this experiment is to test the feasibility to detect drowsiness when and if it occurs by testing the accuracy of locating pupils with sample sequences. In Table 5, TP stands for True Positives, where closed eyes were correctly detected; TN stands for true negatives where opened eyes were correctly detected; FP stands for false positives, where opened eyes were wrongly detected as closed eyes; and FN stands for false negatives, where closed eyes were falsely detected as opened eyes. The accuracy, ACC, is calculated using:

(8) $$\mathrm{ACC}=\frac{\mathrm{TP}+\mathrm{TN}}{\mathrm{Frame}\;\mathrm{Count}}\times 100\%$$

The results show that for these six videos, their accuracies ranges between 88% and 98%, with the average of 94.26%, without validating the eyes while tracking. These results show a good promise that the driver would be alerted when and if drowsiness occurs.

## 5. Conclusions

In this paper, two components to augment the capabilities of vision-based ADAS at night were proposed. We also proposed a modified MSR which would improve the contrast of night-time videos without overexposure and can help improve classifying accuracy. The first component seeks to improve the driver’s safety by alerting the driver when he or she becomes too distracted or drowsy to process warnings. The second component seeks to alert the driver when large vehicles come near the driver’s vehicle. Both components use our modified MSR as a preprocessor. The first component was designed to evaluate driver drowsiness at night by tracking the driver’s eyes and detecting the presence of the pupils using an infrared camera. The second component uses the images from a standard driving video recorder to differentiate vehicles by size. It locates the tail-lights, and use them as hints to extract regions, each of which should contain a single vehicle. In this component, a trained Ada-boost classifier is used to quickly detect large vehicles. The identified large vehicles then are tracked immediately in a CPU process independent of the secondary classifiers which are designed to catch false negatives from the first classifier. The negatives of the first classifier, which are assume to contain false negatives, are first enhanced by a Sigmoid-based MSR before being processed by two types of classifiers, one of which—the KAZE features-based classifier—uses a key-points detection-based approach. The second type—the CNN feature-base classifier—computes a feature vector directly using the entire region. Several experiments were designed to test each stage of the flowcharts of these two components. The results of these experiments together show that the false negatives of the LBP-based Adaboost classifier can be caught by the secondary classifiers. Our experiments also shows that while the CNN-features-based classifier is the preferred classifier if the objects have relatively clean backgrounds, for classifying images containing easy-to-misidentify features, a key-point based classifier like the KAZE BoF can perform better. Also, in order to test the classification accuracy under sub-ideal driving conditions, at least one of the experiments included images taken while driving under bad conditions, such as rainshowers. From the results of these experiments, the classification accuracy results show that these classifiers can be trained to perform their designed classification tasks even under bad driving conditions. Future researches include combining different features into a single classifier and define the interactions between these two components.

## Figures and Tables

**Table 1:** LBP-based Adaboost Classifier Classification Results.

Classifier	Stages	Accuracy (%)
LBP	20	84%
LBP	21	96%

**Table 2:** BoF classifiers classification accuracy results.

Classifier	Bus	Trucks	Trailers	Sedans
KAZE	97.1%	100%	100%	98.14%
AKAZE	96.3%	89.06%	74.86%	94.77%

**Table 3:** Size of feature vector per image.

CNN	HOG	LBP
4096	26,244	46,256

**Table 4:** Average time to process each image.

Time	CNN	KAZE	AKAZE
Feature Extraction time	0.215 s	0.52 s	0.145 s
SVM Training time	17.55 s	28 s	11 s
SVM Testing time	0.02 s	0.0125 s	0.0083 s
Classification time	0.235 s	0.5325 s	0.1533 s

**Table 5:** Results of Detecting Eyes Closed.

Sequence	Frame Count	TP	TN	FP	FN	ACC (%)
1	447	101	332	8	6	96.87
2	333	104	189	14	26	87.98
3	371	209	131	19	15	91.64
4	583	31	541	1	10	98.11
5	535	287	214	25	9	93.64
6	4286	1425	2615	235	11	94.26%
